# Running intralimb coordination patterns after a foot core exercise program in recreational runners

**DOI:** 10.1590/1414-431X2023e13124

**Published:** 2024-01-22

**Authors:** E.Y. Suda, M.F. Vieira, A.B. Matias, R.S. Gomide, I.C.N. Sacco

**Affiliations:** 1 Programa de Pós-Graduação em Fisioterapia, Universidade Ibirapuera, São Paulo, SP, Brasil; 2 Departamento de Fisioterapia, Fonoaudiologia e Terapia Ocupacional, Faculdade de Medicina, Universidade de São Paulo, São Paulo, SP, Brasil; 3 Laboratório de Bioengenharia e Biomecânica, Universidade Federal de Goiás, Goiânia, GO, Brasil

**Keywords:** Running, Exercise therapy, Foot joint kinematics, Coordination patterns

## Abstract

This study investigated the effects of a foot core intervention on the coordination of foot joints in recreational runners. This was a secondary analysis from a randomized controlled trial conducted with 87 recreational runners allocated to the control group (CG), which followed a placebo lower limb stretching protocol, or the intervention group (IG), which underwent an 8-week (3 times/week) foot core training. The participants ran on a force-instrumented treadmill at a self-selected speed (9.5-10.5 km/h) while the foot segment motion was captured. The vector coding technique was used to assess inter-joint coordination for four selected coupled segment and joint angles. The coordination patterns of the calcaneus and midfoot (CalMid) and midfoot and metatarsus (MidMet) joint pairs were affected. In the frontal plane, IG showed an in-phase with proximal dominancy coordination at heel strike, with a decrease in its frequency after the training (P=0.018), suggesting a longer foot supination. Additionally, IG showed an anti-phase with distal dominancy pattern at early stance compared to CG due to a smaller but earlier inversion of the CalMid-MidMet pair (P=0.020). The intervention also had an effect on the transverse plane of the CalMid-MidMet pair, with IG showing a significantly greater frequency of anti-phase coordination with proximal dominancy during propulsion than CG (P=0.013), probably due to a reduction in the CalMid abduction. Overall, the results suggested that the foot core intervention reduces the occurrence of running-related injuries by increasing the resistance to calcaneus pronation and building a more rigid and efficient lever during push-off.

## Introduction

Running has many beneficial effects on musculoskeletal structures (especially for the lower limbs), such as increasing volume and cross-sectional area of foot muscles and increasing bone density (1). However, it also leads to running-related injuries (RRIs) due to its repetitive cyclic loading characteristic. The prevalence of RRIs in the lower limbs during running can be as high as 79.3% in a year of practice (2); thus, preventive measures are crucial for keeping the runner active so that they continue to benefit from the running practice. The etiology of RRIs is multifactorial (3), but it generally includes biomechanical alterations in either the distal or proximal joints, such as altered medial longitudinal arch posture (4), greater rearfoot eversion (5), increased external hip adduction and internal knee rotation moment (6), increased hip internal rotation, and increased knee abduction and external rotation (5,7). The recognition of altered movement patterns as risk factors for RRIs has impelled the development of therapeutic approaches to reduce RRIs. One approach is strengthening the hip and core musculature (abdominal and multifidus muscles) (8) to reduce non-sagittal joint movements and moments and thus reduce the load on adjacent joints in the lower limbs (9,10). Another approach is the strengthening of foot-ankle joints to improve shock absorbance, joint motion and stability, and postural adaptability (11-14).

Several interventions to reduce RRIs, such as warm-up, cool-down, and stretching exercises (15), training programs to gradually increase running volume (16), online educational prevention programs (17), and running shoes advisory programs (18), have yielded lackluster results. However, one intervention based on the bottom-up approach resulted in interesting outcomes related to RRI incidence. An 8-week foot core strengthening program for healthy recreational runners resulted in an increase in the intrinsic anatomical cross-sectional area of the foot muscle, an increase in the propulsive impulse during running (11), and a significant 2.42-fold reduction in RRI incidence at the 1-year follow-up compared with a placebo stretching program (14). Matias et al. (19) showed that the foot core exercise program was also capable of changing the foot-ankle kinematic patterns. Individuals who received the intervention showed a more inverted calcaneus and less dorsiflexed midfoot at foot strike, a running pattern at midstance with a less plantar-flexed and more adducted forefoot and a more abducted hallux, and a less dorsiflexed midfoot and less adducted and more dorsiflexed hallux at push-off. The program also resulted in a decreased medial longitudinal arch excursion and increased rearfoot inversion during the stance phase.

Although foot core training clearly changed the kinematic pattern of the longitudinal arch, ankle, tarsometatarsal, midtarsal, and metatarsophalangeal joints, the foot joints form a dynamic interconnected structure that moves in a coordinated manner, where the motion of one segment interacts with the next segment's kinematics, changing the foot mechanics as a whole. Alterations in the motion of the distal foot joints might result in a corresponding compensation in the adjacent joints within the segment or nearby to efficiently perform the running task, leading to a distinct coordination pattern of the contiguous segments. The coordination between segments, flexibility, strength, and adaptability are essential capabilities for producing a functional and efficient movement pattern (20). Running is a cyclic activity that depends on a functional and efficient coordination of distal and proximal joints of the lower limbs, which in turn depends on and results in an appropriate foot strike/contact with the ground at each step of the runner. The presence of an altered coordination pattern might lead to excessive loads in the musculoskeletal structures, increasing the risk of RRIs (21,22). Specifically for the foot-ankle complex, it has been suggested that the presence of an anti-phase coordination pattern (the segments rotate in opposite directions) between the rearfoot and forefoot may result in excessive tension and torsion of the planta fascia tissue that may be related to plantar fasciitis development (23). Furthermore, the study of the coordination patterns adds information to the understanding of the kinematic profiles that cannot be explained by the analysis of kinematic time series or discrete variables. For instance, a study (23) verified that fatigue of the tibialis posterior, an important rearfoot invertor, did not change the discrete rearfoot (eversion peak and excursion) and forefoot (excursion, dorsiflexion, abduction) kinematic variables. However, when the authors reanalyzed the data in terms of coordination patterns, a disruption in the typical coordination between shank, rearfoot, and forefoot after the fatigue protocol was found (24), which might explain the etiology of tibialis posterior injury. Considering that coordination is achieved via the interaction of intrinsic and extrinsic muscle acting across several joints simultaneously (25), by strengthening the foot core muscles through the proposed intervention, the foot joint coordination patterns might also have been positively changed, supporting the 2.42-fold reduction in RRI incidence (26).

Therefore, understanding how the movement and coordination patterns of the foot and ankle accommodate the loads from the foot-ground interaction and how the foot-ankle joints are altered by therapeutic intervention could contribute to further development and implementation of RRI preventive strategies focused on more distal segments. For the evaluation of coordination, nonlinear techniques such as vector coding are used to quantify coordination through the coupling between segments or joints at each time instance, providing information about the dominance of one segment over another, i.e., when one segment rotates at a greater extent or faster than the other segment. The classification of coordination patterns combines phase dominancy (in-phase or anti-phase) and segmental dominancy (distal or proximal) that highlights differences in the segment motion during the running cycle (27). Thus, our aim was to reanalyze data from Matias et al. (19) to quantify the coordination pattern of the foot joints of recreational runners who underwent foot core muscle training using the vector coding technique. As the nature of the intervention emphasized the strengthening of the intrinsic muscles of the foot, we hypothesized that the intervention induces positive changes in the coordination patterns that reflect a different interaction of foot-ankle joint pairs during running stance phase.

## Material and Methods

This study is a secondary analysis of a 12-month randomized single-blinded parallel controlled trial designed to investigate the benefits of a foot core muscle training program on RRI incidence in recreational middle- and long-distance runners. The trial was prospectively registered at ClinicalTrials.gov (Identifier NCT [NCT02306148]; November 28, 2014, under the title [“Effects of Foot Strengthening on the Prevalence of Injuries in Long Distance Runners“]). A detailed description of the study protocol following the CONSORT recommendations was published elsewhere (13).

### Participants and recruitment

Participants were recruited between August 2015 and August 2017 through digital social media advertising and word of mouth. Eligibility criteria included: middle- and long-distance recreational runners between 18 and 55 years old who had been running for at least 1 year, ran between 20 and 100 km per week with no RRI in the 2 months prior to baseline assessment, no experience running barefoot or in minimalist shoes, and without symptoms suggestive of chronic diseases or impairments that could influence running performance (e.g., osteoarthritis). Participants signed an informed consent form approved by the Ethics Committee of the School of Medicine of the University of São Paulo (18/03/2015, Protocol # 031/15). The main researcher (A.B. Matias) explained to each eligible participant every step of the assessment and follow-up, possible risks, and that no compensation or benefits were to be expected.

A sample of 119 runners was randomly allocated to the intervention group (IG) or control group (CG) using a numeric sequence after baseline assessment. A sequence of 119 potential participants was generated into blocks of four to eight people per block. The codes for the groups were kept in opaque, sealed envelopes numbered 1 to 119, and the researchers involved in the allocation and assessments were blinded to the group codes and block size. After the runner had agreed to participate in the study, an independent researcher also blinded to the codes performed the allocation. Data from all participants were kept confidential before, during, and after the study by encoding their names. From the 119 participants included in the full RCT to evaluate RRI incidence over a 1-year follow-up, only 87 participants had their running biomechanics fully assessed and were thus included in the current secondary analysis: 41 in the IG and 46 in the CG. We included 87 participants based on the availability of the whole time series of foot-ankle kinematic data containing at least 10 step cycles in the baseline assessment. The participants’ characteristics at baseline are shown in Table 1.

**Table 1:** Significant changes found for CalMid-MidMet joints in the frontal and transverse planes.

Coordination pattern	Pair of joints	
	CalMid-MidMet frontal	CalMid-MidMet transverse
IPPD 0°-45°	Group and interaction effectsIG Pre > IG Post	NS
IPDD 45°-90°	NS	NS
APDD 90°-135°	Interaction effectCG Post < IG Post	NS
APPD 135°-180°	NS	Group and interaction effectsCG Post < IG Post
IPPD 180°-225°	NS	NS
IPDD 225°-270°	NS	NS
APDD 270°-315°	NS	NS
APPD 315°-360°	NS	NS

CalMid: calcaneus and midfoot; MidMet: midfoot and metatarsus; IPPD: in-phase with proximal dominancy; IPDD: in-phase with distal dominancy, APPD: anti-phase with proximal dominancy; APDD: anti-phase with distal dominancy; CG: control group; IG: intervention group; NS: not significant.

### Foot core intervention

Participants in the IG received 8 weeks of foot core muscle training containing 12 exercises that were increased weekly in volume and difficulty. The training was performed once a week with a physiotherapist and three other times a week under remote supervision by the same physiotherapist following online exercise descriptions and videos (web software). CG participants were instructed to perform a 5-min static stretching placebo protocol three times a week based on online descriptions (web software) and images.

### Biomechanical assessment

The assessment consisted of two evaluations: at baseline and after 8 weeks. Foot-ankle kinematics data were collected using eight infrared cameras (Vicon^®^ VERO, Vicon Motion Systems Ltd., UK) at 200 Hz with 16 reflective skin markers (9 mm in diameter) placed according to the Rizzoli foot model (28). Following a standing calibration trial, the participants were requested to run barefoot at a self-selected comfortable speed on an instrumented treadmill (AMTI Force-Sensing Treadmill; AMTI, USA) with no incline. Two force plates were embedded in the treadmill in a tandem position. Participants ran barefoot on the treadmill to allow placement and tracking of foot markers. Although this is an unusual condition for participants, their usual foot strike pattern was confirmed by visual inspection of high-speed videos (125 fps) capturing force plate contact in the sagittal plane. Stance phase was defined as the time interval between foot contact and “toe off”. A threshold of 10 N in the vertical ground reaction forces was used to determine foot contact and toe off events.

Before data acquisition, participants went through a habituation period to get used to the laboratory environment and to ensure appropriate running speed. Participants ran on the treadmill at a comfortable speed for 3 min to warm up. Following the warm-up, speed was increased by the participant to a comfortable training pace, ranging from 9.5 to 11 km/h and monitored by the treadmill controls. Kinematic data were recorded for 30 s to acquire at least 10 step cycles for each limb. Only data from the dominant limb, which was determined as the one with which the subject would kick a ball, were used in the analysis.

Kinematic data were filtered using a fourth-order, zero-lag, low-pass Butterworth filter with a cut-off frequency of 10 Hz. The outputs of the Rizzoli foot model were calculated using custom-made scripts in Visual3D (C-Motion, USA) in accordance with the published definitions (28). The multisegmental foot model utilized in this study also followed the joint coordinate system according to the International Society of Biomechanics recommendations (29). Tri-planar joint rotations were calculated between shank and calcaneus (ShaCal), calcaneus and midfoot (CalMid), midfoot and metatarsus (MidMet), and metatarsus and hallux (MetHal) as relative motion of distal segments with respect to proximal segments. Plantarflexion, adduction, and inversion rotations were negative according to the chosen direction of the axes of the joint coordinate systems. Data were normalized to 0-100% of the stance phase.

### Vector coding analysis

The vector coding technique consists of calculating the coupling angle (*γ*) between different body segments or joints (30). The coupling angle is the orientation of the vector connecting two adjacent points in an angle *vs* angle plot. Due to its directional characteristic, the vector coding technique uses circular statistics to calculate the mean coupling angle.

Briefly, the coupling angle is calculated as the angle of a vector connecting consecutive data points (*i*) in a phase space reconstructed using distal and proximal joint angles:

$$\gamma_{i}=\tan^{-1}\left(\frac{\theta_{D}(i+1)-\theta_{D}(i)}{\theta_{P}(i+1)-\theta_{P}(i)}\right).\frac{180}{\pi }with\theta_{P}(i+1)-\theta_{P}(i)>0$$ (Eq. 1)

$$\gamma_{i}=\tan^{-1}\left(\frac{\theta_{D}(i+1)-\theta_{D}(i)}{\theta_{P}(i+1)-\theta_{P}(i)}\right).\frac{180}{\pi }+180with\theta_{P}(i+1)-\theta_{P}(i)<0$$ (Eq. 2)

where 0≤ γ ≤360° is the coupling angle, *i* represents the consecutive samples in a normalized gait cycle, and γ*_i_* is calculated based on the distal joint angles θ*_D_* and proximal joint angles θ*_P_*.

To avoid coupling angle with indeterminate values, the following conditions were considered:

$$\gamma =\{\begin{array}{c} 90^{\circ }if\theta_{P}(i+1)-\theta_{P}(i)=0\text{and}\theta_{D}(i+1)-\theta_{D}(i)>0\\ -90^{\circ }if\theta_{P}(i+1)-\theta_{P}(i)=0\text{and}\theta_{D}(i+1)-\theta_{D}(i)<0\\ 180^{\circ }if\theta_{P}(i+1)-\theta_{P}(i)<0\text{and}\theta_{D}(i+1)-\theta_{D}(i)=0\\ undefinedif\theta_{P}(i+1)-\theta_{P}(i)=0\text{and}\theta_{D}(i+1)-\theta_{D}(i)=0 \end{array}$$ (Eq. 3)

As the estimated value for the coupling angle must be 0° *γ* 360°, the coupling angle must be corrected if its value is less than 0. In this case, 360° must be added to the γ*_i_*. For an individual (*n*) and for a group, γ*_i_* was calculated from the horizontal ( $\bar{x}$ ) and vertical ( $\bar{y}$ ) components along multiple cycles of gait *j* for each percentage *i* of the gait cycle, taking the average of the cosine of γ*_ji_* for $\bar{x}$ and the average of the sine of γ*_ji_* for $\bar{y}$ :

$$\bar{x_{i}}=\frac{1}{n}\sum_{j=1}^{n}\left(\cos\gamma_{ji}\right)$$ (Eq. 4)

$$\bar{y_{i}}=\frac{1}{n}\sum_{j=1}^{n}\left(\text{sen}\gamma_{ji}\right)$$ (Eq. 5)

Then, the length of the mean coupling vector ( $\bar{r_{i}}$ ) is defined by the square root of the sum of the squared horizontal and vertical components:

$$\bar{r_{i}}=\sqrt{\bar{x_{i}^{2}}+\bar{y_{i}^{2}}}$$ (Eq. 6)

According to the *γ* values, coordination was classified into in-phase with proximal dominancy when 0° ≤ γ*_i_* ≤ 45° or 180° < γ*_i_* ≤ 225°, in-phase with distal dominancy when 45° < γ*_i_* ≤ 90° or 225° < γ*_i_* ≤ 270°, anti-phase with distal dominancy when 90° < γ*_i_* ≤ 135° or 270° < γ*_i_* ≤ 315°, and anti-phase with proximal dominancy when 135° < γ*_i_* ≤ 180° or 315° < γ*_i_* ≤ 360° (31). Coordination pattern and coupling angle were calculated between pairs of joint angles in the same plane. Each coordination pattern occurring in the running cycle was quantified using frequency plots to understand the most prevalent patterns. The frequency bars in the figures present the average occurrences (within each group) of the coupling angle within each coordination pattern, shown as horizontal white and gray segments in the figures.

### Statistical analysis

All data had normal distribution (Kolmogorov-Smirnov test, P>0.05). Repeated measures ANOVA with two factors (groups and assessments) was used to compare the frequency of coordination patterns found in the analysis between joints' pairs in the same plane. *Post hoc* tests with Bonferroni correction were applied when there were significant main effects or/and interaction effects. Statistical analysis was performed using JASP software, version 0.17.1, with a significant level set at α<0.5.

## Results

**Figure 1:**
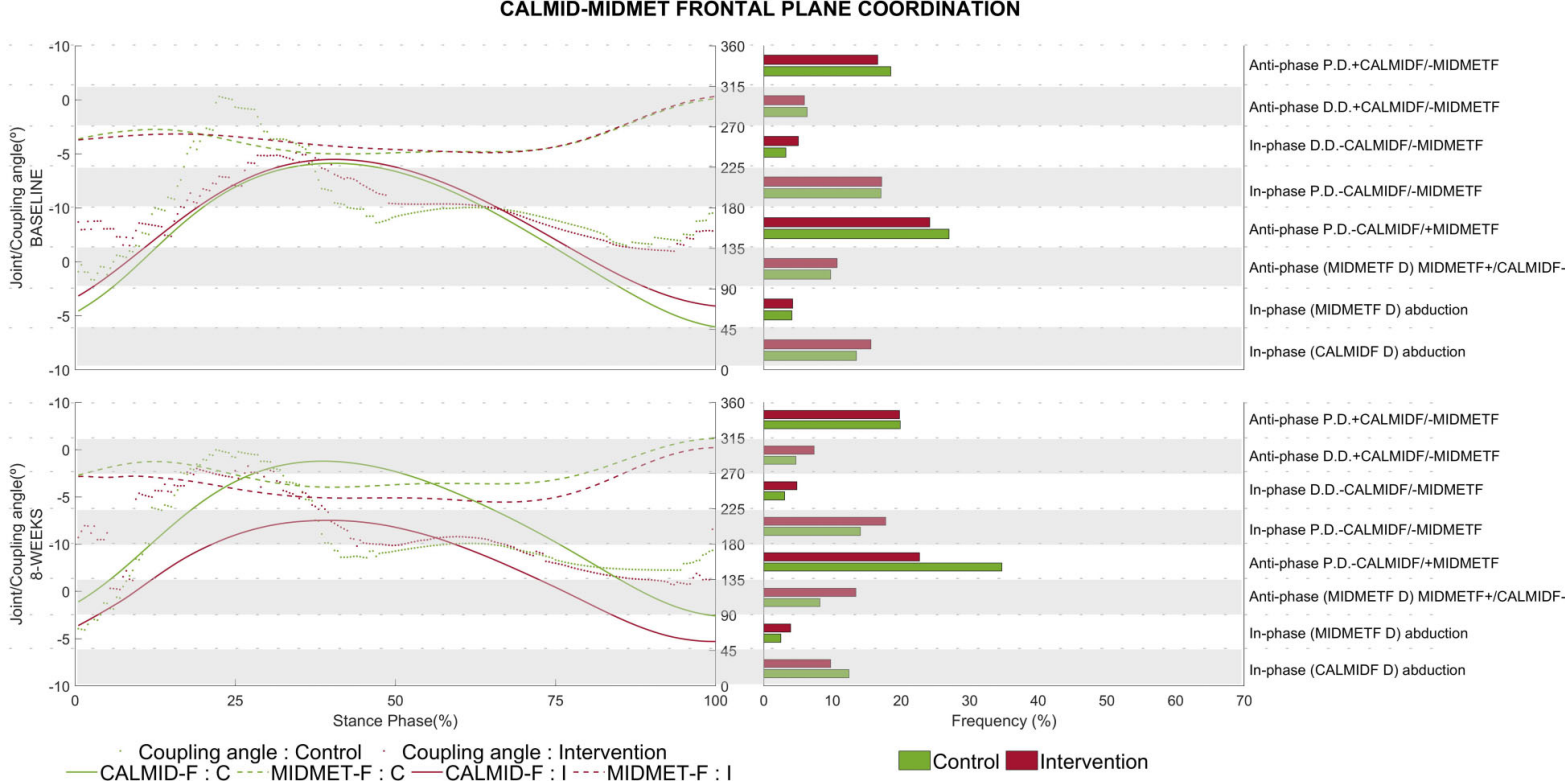
CalMid-MidMet angular displacement diagram (left axis) and frequency of coordination patterns (right axis) in the frontal plane for the control group (CG) and intervention group (IG). The green and red solid lines represent angular displacement of the CalMid joint in the CG and IG, respectively. The green and red dashed lines represent angular displacement of the MidMet joint in the CG and IG, respectively. The green and red dots represent the coupling angle for the CG and IG, respectively. The green and red bar chart represents the average frequency percentage within each group (CG and IG, respectively) of the coupling angle during the gait cycle within each coordination pattern, shown as horizontal white and gray segments. CalMid: calcaneus and midfoot; MidMet: midfoot and metatarsus.

Significant differences in the CalMid-MidMet pairs were found only in the frontal and transverse planes. The other angular combinations can be viewed in the Supplementary Figures S1-S9. For the CalMid-MidMet pair in the frontal plane, significant main group (P<0.001) and interaction (P=0.018) effects were found for the IPPD 0-45° coordination pattern (Figure 1; Table 2), in which the IG showed significantly lower IPPD coordination frequency at loading response phase (0-10%) after the intervention compared to the CG (*post hoc* test, P<0.001). In addition, a significant interaction effect was found for the APDD 90-135° coordination pattern (P=0.020), with an increased frequency for the IG after the intervention compared to the CG (*post hoc* test, P=0.049), mainly during early stance (10-20%) and late propulsion (85-90%).

**Table 2:** Baseline characteristics of participants from the intervention and control groups.

	All participants		Intervention group		Control group	
	n	%/Mean (SD)	n	%/Mean (SD)	n	%/Mean (SD)
n	87	100%	41	47.1%	46	52.9%
Demographics						
Gender (male)	42	48.8%	17	41.5%	25	54.3%
Age (years)		40.3 (6.9)		40.3 (7.7)		40.3 (6.1)
Body mass (kg)		70.5 (13.1)		67.2 (12.1)		73.5 (13.0)
Height (m)		169.3 (8.8)		166.5 (7.6)		171.8 (9.0)
Body mass index (kg/m^2^)		24.5 (3.2)		24.1 (3.0)		24.8 (3.3)
Training						
Running experience (years)		6.5 (5.7)		5.9 (5.1)		7.1 (6.2)
Running frequency per week		3.7 (1.0)		3.8 (1.0)		3.6 (1.2)
Running volume per week (km)		35.8 (27.6)		31.7 (22.5)		39.4 (30.8)
Average pace (min/km)		6.58" (1.36)		6.46" (2.36)		6.69" (2.38)
Running event						
Member of athletic association (yes)	38	43.7%	19	46.3%	19	41.3%
Participated in a running event before (yes)	83	95.4%	40	97.6%	43	93.5%
Number of running events before		37.0 (41.7)		29.3 (31.8)		44.0 (47.5)
Anthropometrics						
Foot posture index, median (25th and 75th percentiles)		2.0 (-2.25; 4.0)		2.0 (-3.0; 4.0)		1.0 (-1.0; 4.0)
Cavanagh & Rodgers arch index (right foot)		0.20 (0.06)		0.22 (0.05)		0.18 (0.07)
Previous RRI in previous 12 months (yes)	40	46.0%	20	48.8%	20	43.5%

RRI: running-related injury.

**Figure 2:**
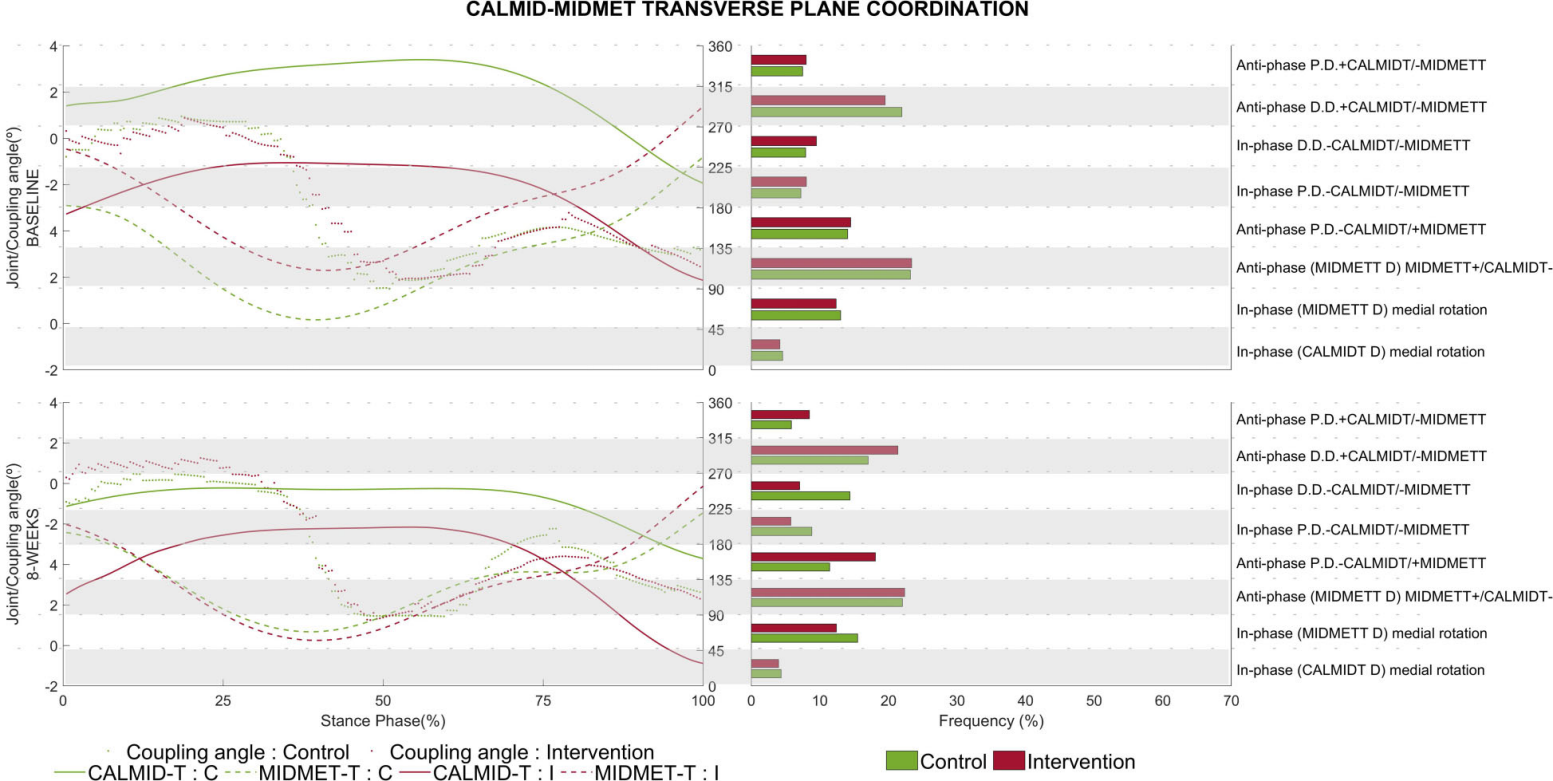
CalMid-MidMet angular displacement diagram (left axis) and frequency of coordination patterns (right axis) in the transverse plane in the control group (CG) and intervention group (IG). The green and red solid lines represent angular displacement of the CalMid joint in the CG and IG, respectively. The green and red dashed lines represent angular displacement of the MidMet joint in the CG and IG, respectively. The green and red dots represent the coupling angle for the CG and IG, respectively. The green and red bar chart represents the average frequency percentage of the coupling angle within each group (CG and IG, respectively) during the gait cycle within each coordination pattern, shown as horizontal white and gray segments. CalMid: calcaneus and midfoot; MidMet: midfoot and metatarsus.

For the CalMid-MidMet pair in the transverse plane, a significant main group effect (P=0.017) and an interaction effect (P=0.013) were found for the APPD 135-180° coordination pattern (Figure 2; Table 2), with an increased frequency for the IG (*post hoc* test, P=0.004) after the intervention compared to the CG, mainly during the propulsion phase (75% stance).

## Discussion

The main purpose of this study was to verify the effects of a foot core intervention on the coordination of the foot joints of recreational runners using the vector coding technique. The results of this study showed that the proposed intervention had an effect on the coordination patterns of the CalMid-MidMet joint pairs in the frontal and transverse planes that might represent a positive adaptation of the foot-ankle complex to the intervention, either during heel strike, early stance, or late propulsion, as hypothesized.

Specifically, in the frontal plane, the CalMid-MidMet pair of joints showed an in-phase with proximal dominancy coordination pattern at the loading response phase (0-10%) showing that the foot is inverted. After an 8-week foot-core training, the IG showed a decrease in this pattern compared to the CG. This pattern was due to a decreased CalMid angle and motion in relation to the MidMet, with the profiles also showing a decreased MidMet angle variation due to the intervention protocol, showing that the foot is still inverted but less mobile as a set. This coordination pattern might be reflecting a more stable foot-ankle complex at heel strike after the intervention protocol.

In addition, also in the frontal plane and at early stance (10-20%), the CalMid-MidMet pair presented an anti-phase with distal dominancy pattern, with the IG presenting an increase in the frequency pattern compared to the CG after 8 weeks due to a smaller but earlier inversion of the CalMid in relation to MidMet. Structurally, the foot can be considered a twisted plate with its anterior portion (the metatarsal heads) horizontally oriented and its posterior portion (the calcaneus) vertically oriented (32). The coordination pattern observed reflects a more twisted foot at early stance that may cause further twisting of the osteoligamentous plate, increasing the resistance to pronation that occurs in the running loading phase, which untwists the plate. This increased resistance to calcaneus pronation in the IG may have provided the necessary protection for the tibiotalar joint from the high traction forces imposed by the evertor and invertor muscles during the stance phase (33), which could have contributed to a lower occurrence of RRIs in the IG, as reported previously (11). A more everted heel at toe off and longer duration of foot eversion during the loading phase have been associated with both Achilles tendinopathy and medial tibial stress syndrome in runners (34).

An anti-phase coordination pattern with distal dominancy in the frontal plane of the CalMid-MidMet joint pair presented a higher frequency at late propulsion (85-90%) for the IG compared to the CG after 8 weeks. Thus, the MidMet joint exhibited a greater excursion than the CalMid at late propulsion after the intervention. This pattern may be beneficial for propulsion during running because it reflects a more supinated foot and, in particular, a rearfoot inversion at late stance is directly linked to locking of the transverse joints (35) and thus building a more rigid and efficient lever during push-off (32,36). Foot core training might have promoted the strengthening of the extrinsic foot-ankle muscles, such as the tibialis posterior, enhancing rearfoot inversion (37). In a previous proof-of-concept study, this foot core program increased the intrinsic anatomical cross-sectional area of the foot muscle and the propulsive impulse during running (14). There was also a significant correlation between time-to-injury and foot strength gain, which could support the hypothesis we described: the stronger the runner's foot, the longer it took the runner to develop an RRI and more efficient running pattern.

The intervention also had an effect on the transverse plane of the CalMid-MidMet pair of joints showing an anti-phase coordination with proximal dominancy with a significantly greater frequency of this pattern during propulsion phase (75% stance) for the IG compared to the CG (Figure 2). After 8 weeks of intervention, the reduction in MidMet segmental dominance during running appears to be the primary factor contributing to this change. There was an earlier reduction in the CalMid abduction after the intervention in the propulsion and a reduction in the MidMet abduction. The alignment of the midfoot and the forefoot in the transverse plane might be reflected in the conformation of the transverse arch. A higher curvature of the transverse arch is associated with greater stiffness of the longitudinal arches, which has an important role during locomotion, especially in providing resistance to the bending in the sagittal plane, enhancing propulsion (38). One could infer that if the intervention promoted a reduction in the CalMid abduction, the foot would be more supinated at the end of late stance (propulsion) and would present a higher transverse arch; thus, the intervention supposedly contributed to foot stiffness during propulsion.

There are some limitations in this study that need to be addressed. Runners were assessed while running barefoot in controlled laboratory conditions, which is different from their outdoor regular shod running practice. As footwear restricts foot-ankle movements and we assessed runners barefoot, the results here should be interpreted cautiously regarding shod running, although this caution may or may not apply to the interpretation of injury risk. However, it would be very difficult to perform the same biomechanical foot-ankle assessment under real-life conditions. In addition, although treadmill running results in lower-limb kinematic patterns similar to overground running (39), this condition may have added some bias to the results. Furthermore, as the coordination analysis was based on skin-marker kinematics of foot segments, this approach might have introduced some errors when estimating out-of-sagittal plane motions of the foot joints, especially during running.

In summary, this study suggested that the foot core intervention presented effects on the coordination patterns of the CalMid-MidMet joint pairs in both the frontal and transverse planes of recreational runners after 8 weeks of training. Specifically, the intervention led to a decrease in the CalMid angle and motion and a decreased MidMet angle variation, resulting in longer and greater rearfoot supination at heel strike. The training also resulted in a more supinated foot at late stance, an earlier reduction in the CalMid abduction, and a reduction in the MidMet abduction during propulsion, which might contribute to greater stiffness in the transverse arch and forefoot, enhancing the push-off during running. These findings suggested that foot core training can be a beneficial intervention for runners, potentially reducing the risk of running-related injuries, as previously shown (26), and improving running coordination of the distal foot joints. Further research in this area may be warranted to explore the potential clinical applications of these findings.

## Supplementary Material

Click to view [pdf].
