# Supplementary material for: Running intralimb coordination patterns after a foot core exercise program in recreational runners
Source: Braz J Med Biol Res. 2024 Jan 22;57:e13124. doi: 10.1590/1414-431X2023e13124 (PMC10802229; doi:10.1590/1414-431X2023e13124)

Figure S1. CalMid-MidMet angular displacement diagram (left axis) and frequency of coordination patterns (right axis) in the sagittal plane in control group (CG) and intervention group (IG). The green and red solid lines are angular displacement of CalMid joint in control and intervention groups, respectively. The green and red dashed lines are angular displacement of MidMet joint in control and intervention groups, respectively. The green and red dots are the coupling angle in CG and IG, respectively. The green and red bar chart represents the average frequency percentage of the coupling angle within each group (CG and IG, respectively) during the gait cycle within each coordination pattern, shown as horizontal white and gray segments. CalMid: calcaneus and midfoot; MidMet: midfoot and metatarsus.

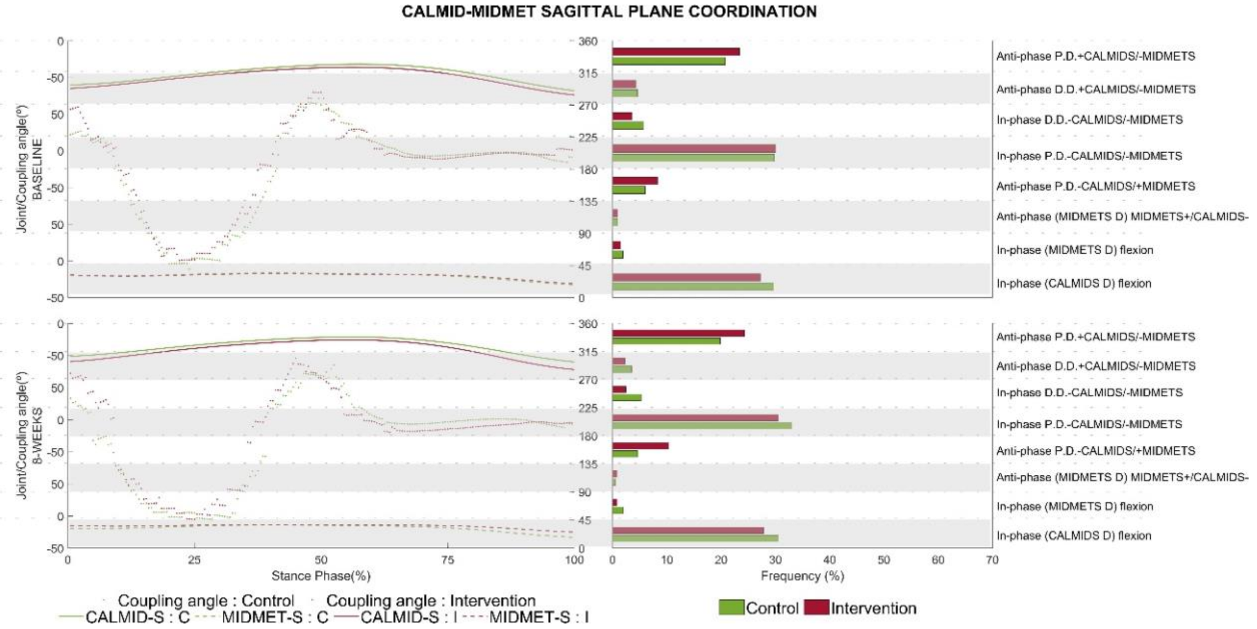

Figure S2. MidMet-MetHal angular displacement diagram (left axis), and frequency of coordination patterns (right axis) in the sagittal plane in control group (CG) and intervention group (IG). The green and red solid lines are angular displacement of MidMet joint in control and intervention groups, respectively. The green and red dashed lines are angular displacement of MetHal joint in control and intervention groups, respectively. The green and red dots are the coupling angle in CG and IG, respectively. The green and red bar chart represents the average frequency percentage of the coupling angle within each group (CG and IG, respectively) during the gait cycle within each coordination pattern, shown as horizontal white and gray segments. MidMet: midfoot and metatarsus; MetHal: metatarsus and hallux.

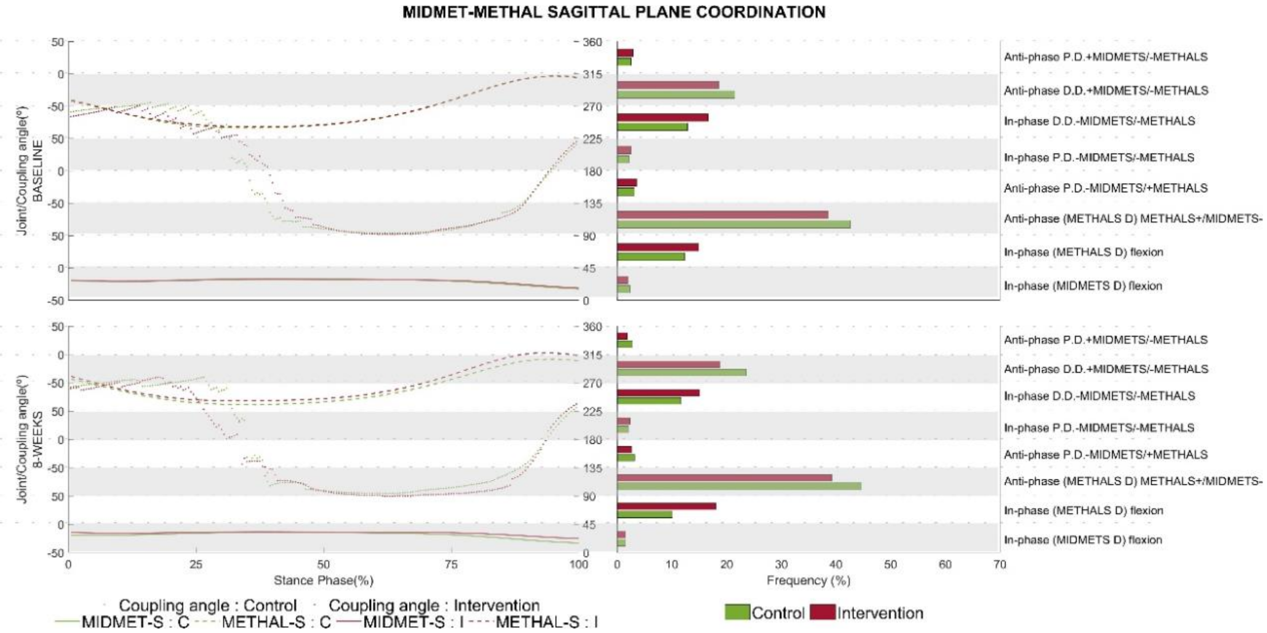

Figure S3. MidMet-MetHal angular displacement diagram (left axis), and frequency of coordination patterns (right axis) in the transverse plane in control group (CG) and intervention group (IG). The green and red solid lines are angular displacement of MidMet joint in CG and IG, respectively. The green and red dashed lines are angular displacement of MetHal joint in control and intervention groups, respectively. The green and red dots are the coupling angle in control and intervention groups, respectively. The green and red bar chart represents the average frequency percentage of the coupling angle within each group (CG and IG, respectively) during the gait cycle within each coordination pattern, shown as horizontal white and gray segments. MetHal: metatarsus and hallux; MidMet: midfoot and metatarsus.

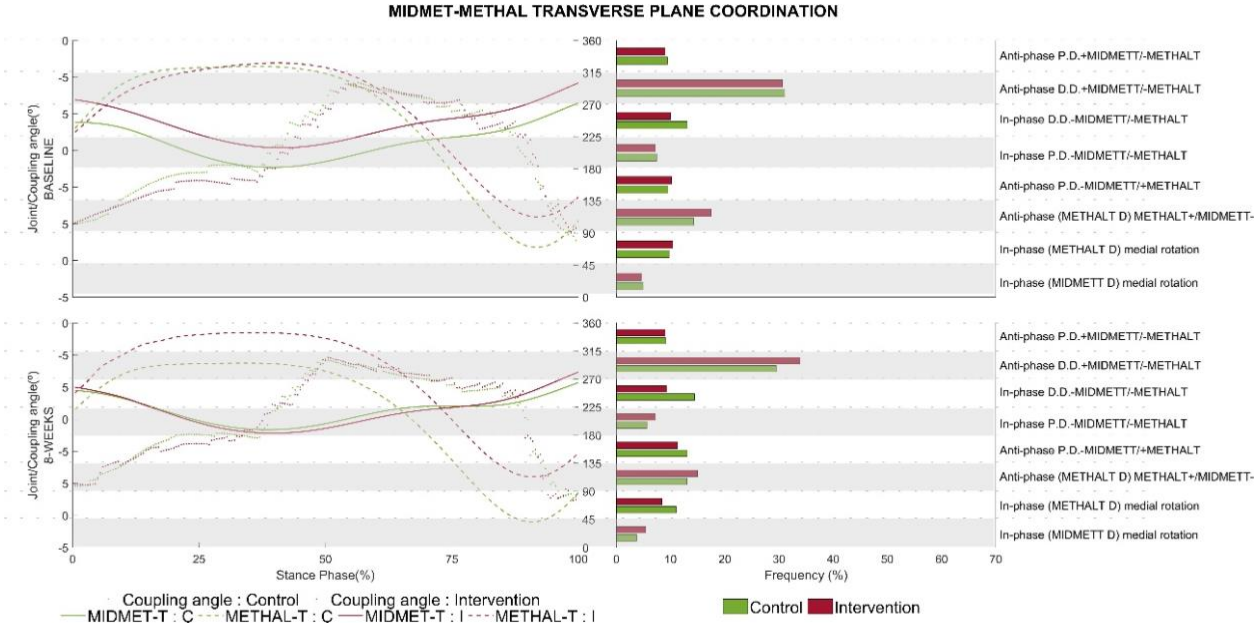

Figure S4. ShaCal-CalMid angular displacement diagram (left axis), and frequency of coordination patterns (right axis) in the frontal plane in control group (CG) and intervention group (IG). The green and red solid lines are angular displacement of ShaCal joint in control and intervention groups, respectively. The green and red dashed lines are angular displacement of CalMid joint in control and intervention groups, respectively. The green and red dots are the coupling angle in control and intervention groups, respectively. The green and red bar chart represents the average frequency percentage of the coupling angle within each group (CG and IG, respectively) during the gait cycle within each coordination pattern, shown as horizontal white and gray segments. ShaCal: shank and calcaneus; CalMid: calcaneus and midfoot.

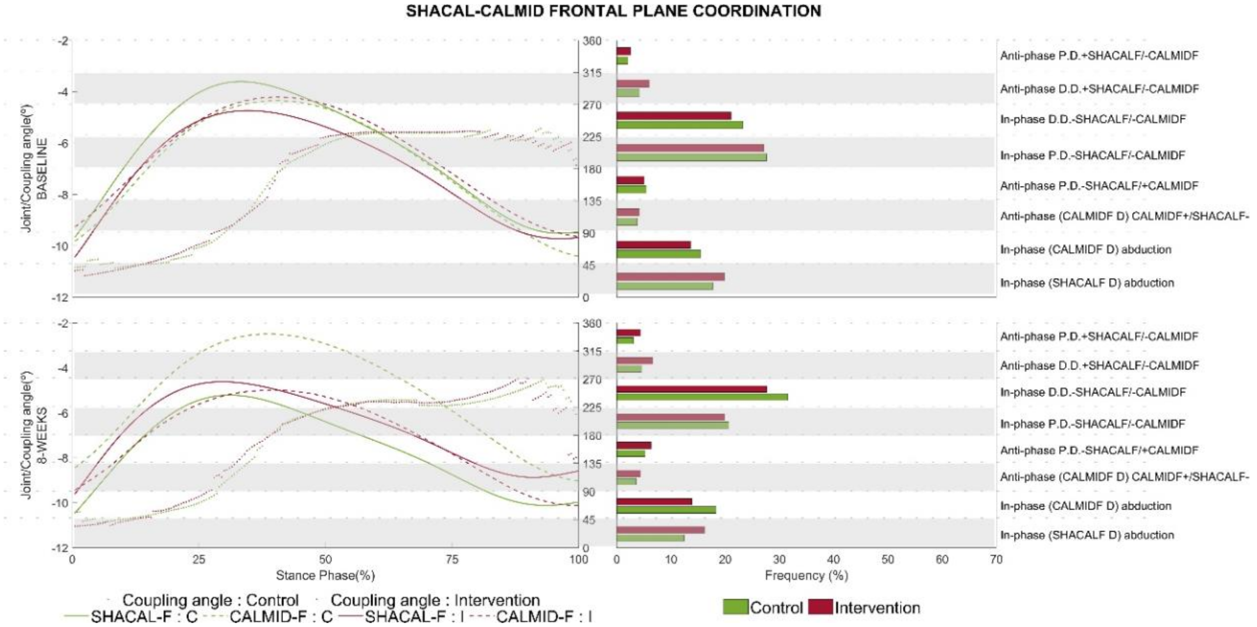

Figure S5. ShaCal-MidMet angular displacement diagram (left axis), and frequency of coordination patterns (right axis) in the frontal plane in control group (CG) and intervention group (IG). The green and red solid lines are angular displacement of ShaCal joint in CG and IG, respectively. The green and red dashed lines are angular displacement of MidMet joint in control and intervention groups, respectively. The green and red dots are the coupling angle in control and intervention groups, respectively. The green and red bar chart represents the average frequency percentage of the coupling angle within each group (CG and IG, respectively) during the gait cycle within each coordination pattern, shown as horizontal white and gray segments. ShaCal: shank and calcaneus; MidMet: midfoot and metatarsus.

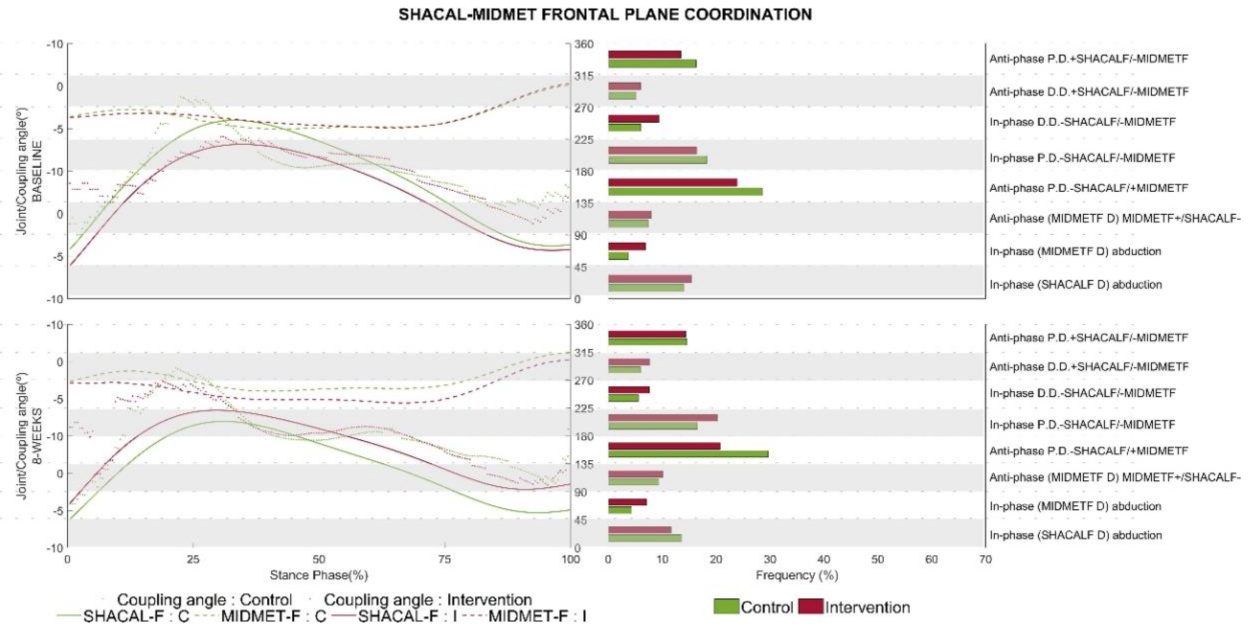

Figure S6. ShaCal-CalMid angular displacement diagram (left axis), and frequency of coordination patterns (right axis) in the sagittal plane in control group (CG) and intervention group (IG). The green and red solid lines are angular displacement of ShaCal joint in CG and IG, respectively. The green and red dashed lines are angular displacement of CalMid joint in control and intervention groups, respectively. The green and red dots are the coupling angle in control and intervention groups, respectively. The green and red bar chart represents the average frequency percentage of the coupling angle within each group (CG and IG, respectively) during the gait cycle within each coordination pattern, shown as horizontal white and gray segments. ShaCal: shank and calcaneus; CalMid: calcaneus and midfoot.

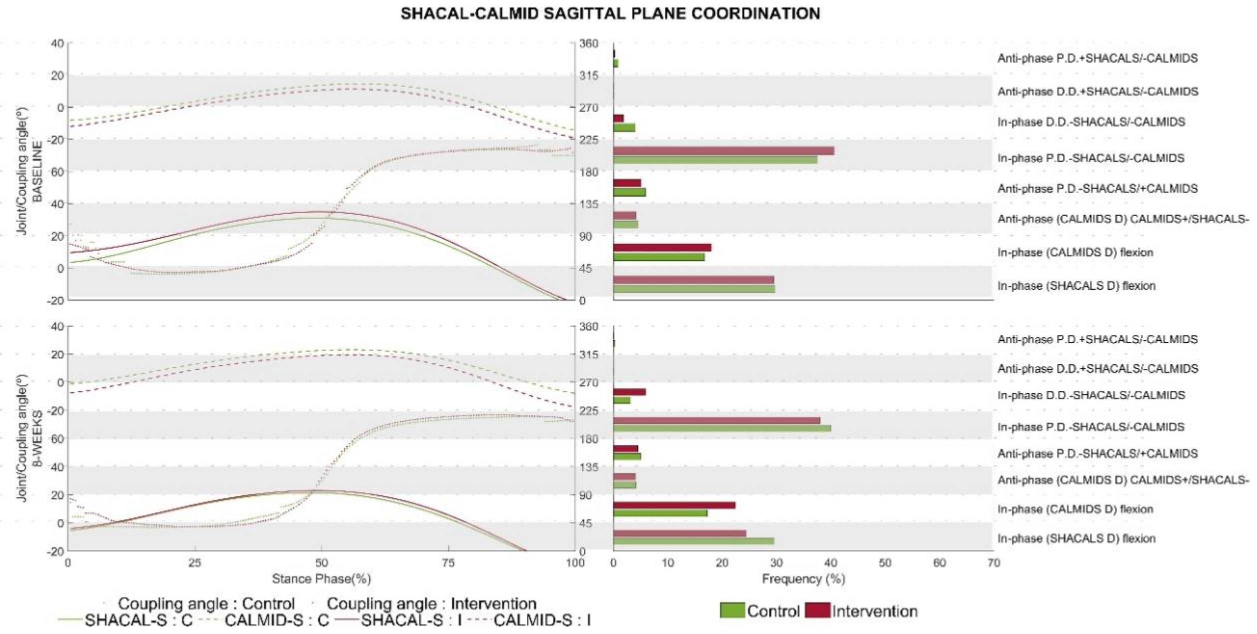

Figure S7. ShaCal-MidMet angular displacement diagram (left axis), and frequency of coordination patterns (right axis) in the sagittal plane in control group (CG) and intervention group (IG). The green and red solid lines are angular displacement of ShaCal joint in CG and IG, respectively. The green and red dashed lines are angular displacement of MidMet joint in control and intervention groups, respectively. The green and red dots are the coupling angle in control and intervention groups, respectively. The green and red bar chart represents the average frequency percentage of the coupling angle within each group (CG and IG, respectively) during the gait cycle within each coordination pattern, shown as horizontal white and gray segments. ShaCal: shank and calcaneus; MidMet: midfoot and metatarsus.

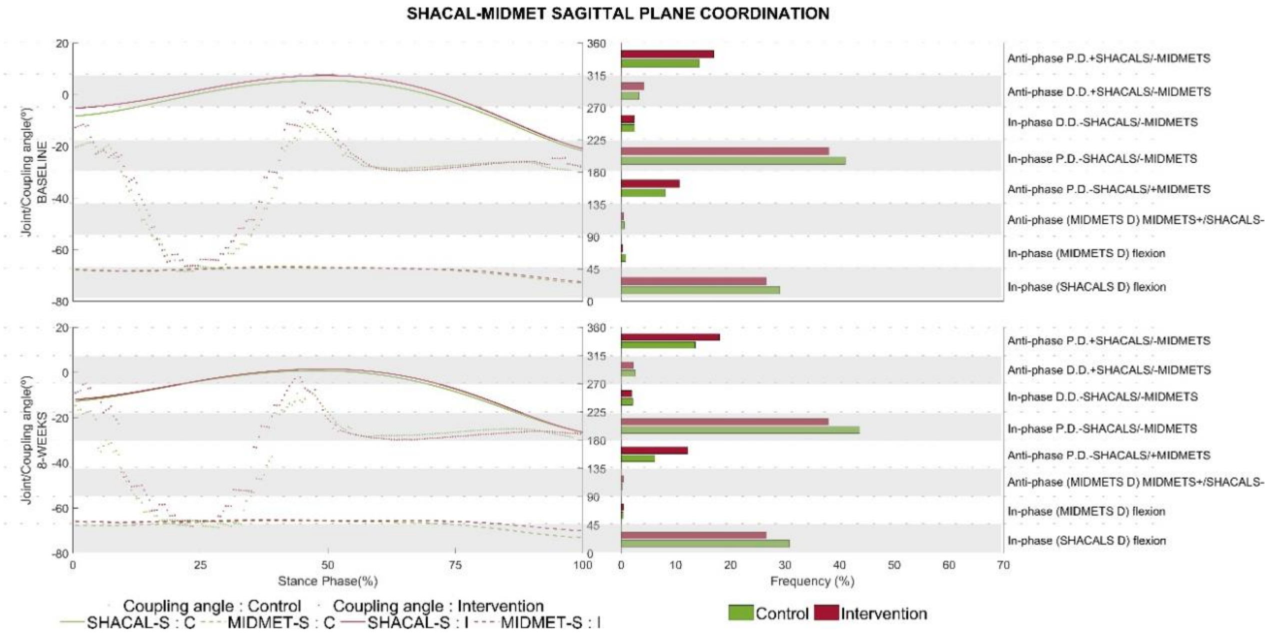

Figure S8. ShaCal-CalMid angular displacement diagram (left axis), and frequency of coordination patterns (right axis) in the transverse plane in control group (CG) and intervention group (IG). The green and red solid lines are angular displacement of ShaCal joint in CG and IG, respectively. The green and red dashed lines are angular displacement of CalMid joint in control and intervention groups, respectively. The green and red dots are the coupling angle in control and intervention groups, respectively. The green and red bar chart represents the average frequency percentage of the coupling angle within each group (CG and IG, respectively) during the gait cycle within each coordination pattern, shown as horizontal white and gray segments. ShaCal: shank and calcaneus; CalMid: calcaneus and midfoot.

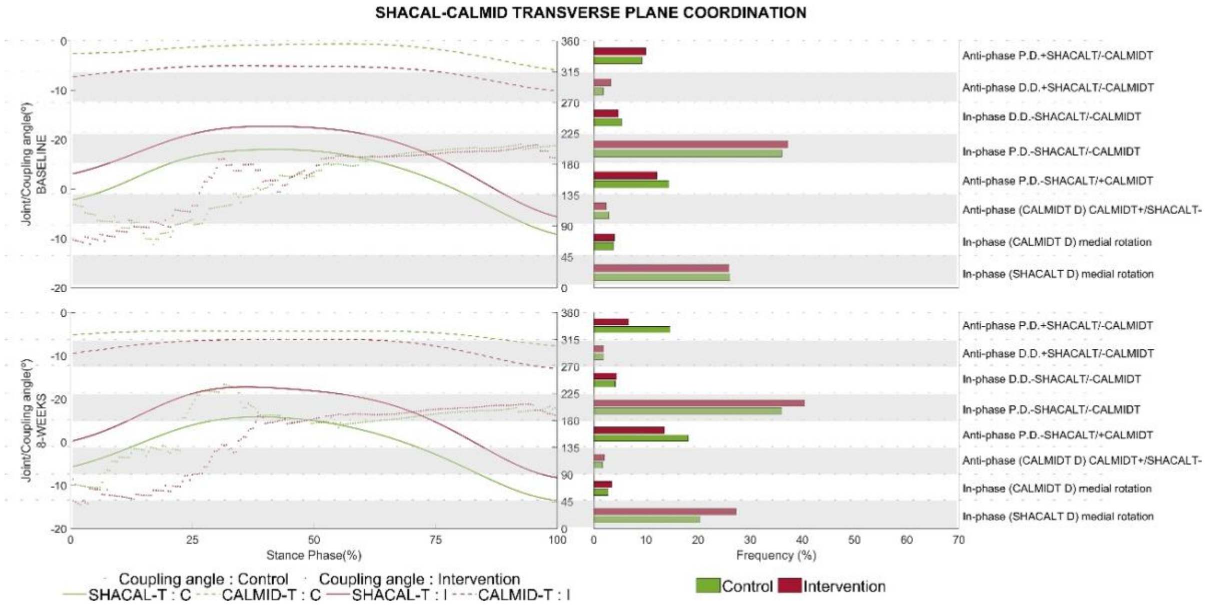

Figure S9. ShaCal-MidMet angular displacement diagram (left axis), and frequency of coordination patterns (right axis) in the transverse plane in control group (CG) and intervention group (IG). The green and red solid lines are angular displacement of ShaCal joint in CG and IG, respectively. The green and red dashed lines are angular displacement of MidMet joint in control and intervention groups, respectively. The green and red dots are the coupling angle in control and intervention groups, respectively. The green and red bar chart represents the average frequency percentage of the coupling angle within each group (CG and IG, respectively) during the gait cycle within each coordination pattern, shown as horizontal white and gray segments. ShaCal: shank and calcaneus; MidMet: midfoot and metatarsus.

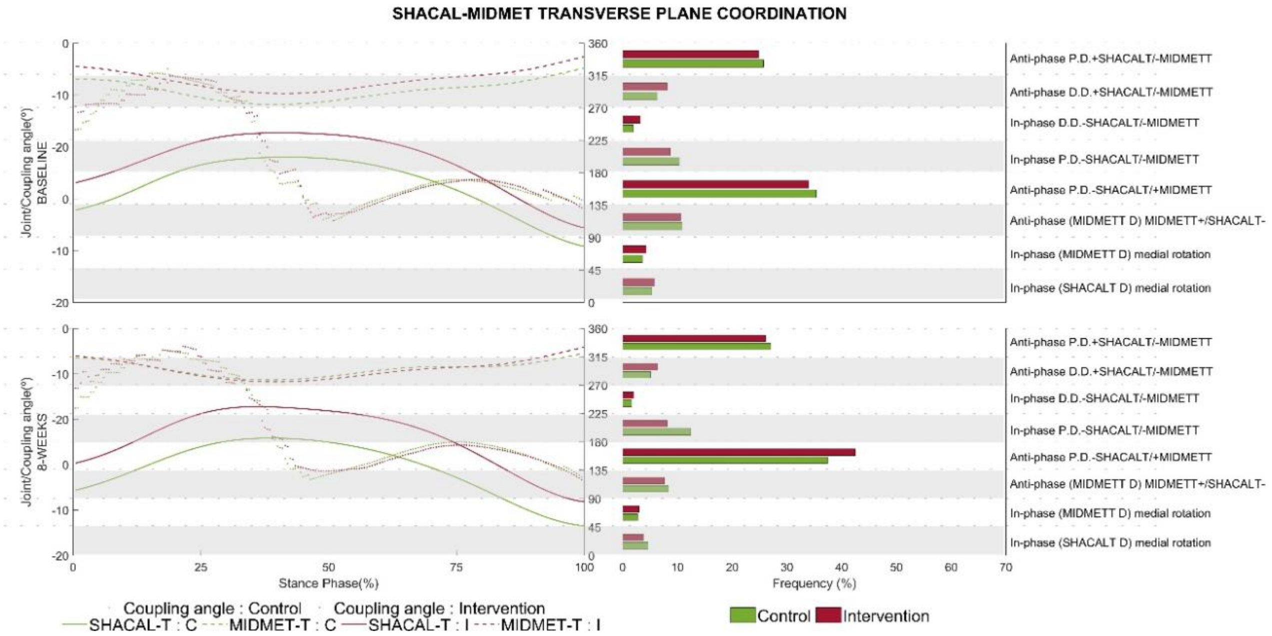

Supplement: Supplementary file 1 [file 1414-431X-bjmbr-57-e13124-suppl.pdf]
